# Data on the demographic forecast of the Kazakhstan population

**DOI:** 10.1016/j.dib.2023.109985

**Published:** 2023-12-18

**Authors:** Lazat S. Spankulova, Zaure K. Chulanova

**Affiliations:** a Al-Farabi Kazakh National University Kazakhstan, 71 Al-Farabi Avenue, Almaty 050040, Kazakhstan; b Institute of Economics of the CS MSHE RK, 28 Shevchenko Str., Almaty 050010, Kazakhstan

**Keywords:** Population, Forecast, Dynamics, Fertility, Migration

## Abstract

The purpose of the study is to forecast the demographic situation in Kazakhstan until 2050 for Kazakhstan and its regions. Forecasts of population size and structure are developed based on analysis of trends in demographic processes, and their cause-and-effect relationships with socio-economic processes. Thus, the calculations take into account the mortality rates observed in 2020–2021 due to COVID-19. The results obtained include data on the total population of Kazakhstan, gender distribution, age structure of the population, data on fertility and mortality (including infant mortality) for the period from 2022 to 2100. The cohort-component method was used, and alternative forecasting methods were proposed. The article presents data on the prospective population of Kazakhstan based on indicators of natural and mechanical population movement, exponential curve, and average growth rate, respectively, for the period 2020–2050.

Specifications Table

**Table:** 

Subject	Social sciences
Specific subject area	Demography
Data format	Analyzed, Forecast data
Type of data	Tables, Charts, Figures
How data were acquired	Statistical data, Analyses, Calculations, Forecasting
Data collection	The data was obtained as a result of a forecast of the population of Kazakhstan for the future. The forecast data includes the total population of Kazakhstan, gender distribution, the age structure of the population, fertility and mortality (including infant mortality) for the period from 2022 to 2100.
Data source location	Republic of Kazakhstan Chulanova, Zaure; Spankulova, Lazat (2023), “Demographic forecast of the population of Kazakhstan”, Mendeley Data, V1, doi:10.17632/nchwcsdsk3.1
Data accessibility	https://data.mendeley.com/datasets/jdvsyzypzk/1 The “Demograph forecast” data in the repository is presented in Excel and contains the results of the demographic analysis and population forecast of Kazakhstan until 2100 (including 8 tables and 3 graphs)

## 1 Value of the Data

The value of the data presented is determined by the following provisions:

• The demographic problem for Kazakhstan is very relevant and especially acute at the present development stage. There are certain problems of demographic development: the demographic situation in Kazakhstan is characterized by an increase in the proportion of older people in the age structure of the country's population, increasing demographic pressure, uneven distribution of the population throughout the country.
• The demographic forecast should form the basis of socio-economic planning for the country's development in the future. Economic, political, and most other social structures need objective and accurate information about the upcoming population, its gender and age structure, etc. This is necessary for plans for the schools, hospitals, kindergartens construction, social welfare planning, and social innovation by region.
• Population statistics were taken as a basis, based on the analysis of actual data, existing development trends, factors influencing it were identified, and a forecast was made. The data obtained are new and forward-looking.
• The forecast was made taking into account the study of current trends in population reproduction by assessing their possible impact on the future size and composition of the population. The forecast shows the realistically possible scale of the future demographic picture, taking into account modern socio-economic factors, for example, the COVID-19 pandemic.
• The main methods used to calculate the prospective population are considered and used, their main advantages and disadvantages are analyzed.
• Experimental calculations were carried out based on actual data and an analysis of the results obtained.
• The conducted research introduces new methods and opportunities for demographic forecasting.

## 2 Data Description

Demographic forecast of the expected size and age and sex structure of the country's population is made based on the actual structure and existing and proposed birth rates at different ages, migration trends.

According to the calculations carried out, the following data were obtained on the future population of Kazakhstan.

Initially, the following data were calculated:  

Total fertility rate;

Total fertility rate;

Birth rate;

Migration balance, thousand people.  

Then, based on the data obtained, a forecast is made:  

Population forecast using a cohort-component forecasting method;

Population forecast by age groups up to 2050; population forecast, scenario “2020” and deviation to “2020”;

Alternative forecasts were also made:  

Population forecast using the average growth coefficient;

Population forecast using exponential curve formulas;

Population forecast using natural and mechanical movements;

Population forecast using second and third-degree polynomials.

The data were processed using the IBM SPSS Statistics program.

## 3 Experimental design, materials and methods

Demographic forecasting is a key tool for understanding and managing population dynamics. The impact of demographic factors on various aspects of a country's development is enormous and includes social, economic and political aspects.

High-quality demographic forecasts are capable of solving a wide range of problems of regional and municipal management.

Forecasts are indispensable when developing demographic and social policy measures and assessing their possible consequences. Qualitative models make it possible to assess the consequences of various socio-economic policy measures and simulate various development scenarios.

Demographic questions not only describe the expected future, but also help to better understand the present. After all, promising changes in the demographic situation are largely based on those phenomena that are already observed today. Any forecast is a look at the present from the point of view of how it is projected into the future.

Forecasting was carried out using data on the Kazakhstan population in dynamics from 1990 to 2022. The calculations were carried out according to the following indicators: Population (thousand people), Number of births, Number of deaths, Natural increase, Crude birth rate (per 1000), Crude death rate (per 1000), Natural increase (per 1000).

The data source used for forecasting is official statistics of the Republic of Kazakhstan. The data are available on the Bureau of National Statistics of Kazakhstan website [1]. The methodology for calculating indicators of number and structure refers to the statistical methodology, formed in accordance with international standards and approved in accordance with the Law of the Republic of Kazakhstan dated March 19, 2010 “On State Statistics”. The main sources of information on the population of the country and its regions are data obtained from national censuses.

Current estimates of the permanent population at the beginning of the year are calculated on the basis of data obtained from the results of the latest national census, given from the date of the national census to January 1 of the census year, to which are added annually the number of births and those arriving for permanent residence in the territory of the Republic of Kazakhstan or its regions and from which the numbers of deaths and those who left for permanent residence outside the Republic of Kazakhstan or its regions are subtracted.

**Fig. 1:**
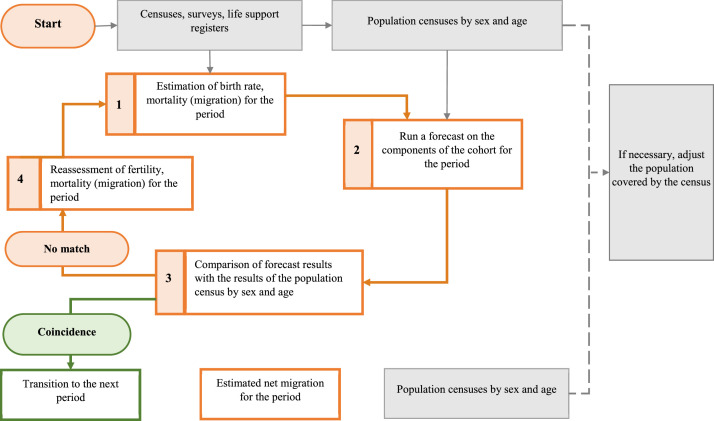
Scheme of estimation of the parameters of the future demographic situation.

Using these data, the authors made a forecast of the population of Kazakhstan for the medium and long term. The calculation methodology and the data obtained are presented in the article. Using the existing models for assessing the parameters of the future demographic situation and the demographic forecast [2], we identified the following main stages for assessing the parameters of the future demographic situation:

- estimation of fertility, mortality, migration for the period;
- making a forecast by cohort components for the period;
- comparison of forecast results with census data by age and gender;
- revaluation of fertility, mortality, migration over the period (Fig. 1).

Demographic forecasting methods include: extrapolation, cohort-component, statistical modeling methods.

The use of extrapolation methods to estimate the future population size is based on the assumption that the identified trends in fertility, mortality, and migration will remain unchanged over the forecast period of time. Those are probabilistic in nature, not taking into account emerging situations such as a decrease in the birth rate and an increase in migration in crisis conditions, high mortality during a pandemic, etc.

The key objective of the cohort-component forecasting method is to ensure that past trends and future changes in fertility, mortality and international migration are consistent for each country [3]. The essence of the method is to divide the population into cohorts by age and sex and subsequent step-by-step changes in the numbers of these cohorts in accordance with age-sex fertility, mortality and migration rates. That part of the cohort that did not migrate and did not die during the step moves to the next age group. Thus, at each modeling step, population aging is simulated. The number of births is determined based on the size of the female population of reproductive age and age-specific birth rates.

Modeling methods are used to solve more complex and specific problems.

For this study, the cohort-component method of demographic forecasting was chosen as the most accurate. An advantage of the cohort-component method is the ability to predict the age-sex structure of the population. Prospective estimates of the population age-sex structure serve as the basis for functional forecasts.

Concerning the parameters of the number of historical years that will serve as the basis for further forecasting, the following conventions can be identified:

- if we take a short period as the basis for the forecast (from 1 day to 1 year), then the forecast should be made for a short period. For example, to predict the behavior of medical services for the year ahead (emergency department).
- if we take the average term, then the forecast should be made for the near future.

To begin work on predicting the future demographic situation, a current estimate of the population is needed.

Current estimates are formed based on continuous accounting: population censuses, data on the registered birth rate, mortality and migration of the population, current accounting of the movement of the population by internal affairs, justice, and health with monthly frequency; sample surveys.

Population estimates are made based on the results of the last population census, to which the number of births and arrivals to this territory is added annually, and the number of deaths and departures from this territory is subtracted.

The forecast using the cohort-component method consists of the following. According to the census data, a certain number of people are registered in this age group S(x, t). After a year, these people will move to the next age group, and they will survive until the next year in a certain ratio, which is taken from the mortality tables in the form of the survival rate P(x) calculated in them. If we multiply the population size in the age group S (x, t) by the corresponding survival rate P (x, t), then the resulting value will characterize the population size a year later in the next age group S (*x* + 1, *t* + 1). To determine the population size for the next years of the forecast, the operation is repeated. The calculation formula is:

$$\begin{array}{c} S(x+1,t+1)=S(x,t)*P(x,t), \end{array}$$

Where S(x, t) - the number of people in a given age group;

P(x, t) - survival rate in the corresponding age group;

S (*x* + 1, *t* + 1) - population size for the year in the next age group.

The expected number of births per year (t) is calculated by multiplying the number of women aged 15–49 by the corresponding fertility rates F(x, t) obtained from fertility tables.

All stages of the demographic forecast are in a causal relationship. The stages follow each other in strict order: the assessment of fertility, mortality, migration over a period allows us to obtain probabilistic parameters of the future demographic situation, which are predetermined in the forecasts for the cohort components for the period, then the results of the forecast are compared with the census data by age and by gender, after which the parameters of fertility, mortality, migration for the period are reassessed.

Calculations of the future population based on alternative methods [4] also given.

## 4 Forecast Data

According to the results given in Tables 1 and 2 (UN methodology), the following conclusions have been obtained regarding some demographic indicators.

**Table 1:** Total fertility rate.

Option	2020–2025	2025–2030	2030–2035	2035–2040	2040–2045	2045–2050	2050–2055	2055–2060	2060–2065	2065–2070	2070–2075	2075–2080	2080–2085	2085–2090	2090–2095	2095–2100
Evaluations	4,41	4,56	4,43	3,67	3,61	3,23	3,04	3,03	2,55	2	2,01	2,54	2,67	2,76		
Middle option	2,62	2,5	2,4	2,31	2,23	2,16	2,1	2,05	2,01	1,97	1,94	1,91	1,89	1,87	1,85	1,84
High option	2,87	2,9	2,9	2,81	2,73	2,66	2,6	2,55	2,51	2,47	2,44	2,41	2,39	2,37	2,35	2,34
Low option	2,37	2,1	1,9	1,81	1,73	1,66	1,6	1,55	1,51	1,47	1,44	1,41	1,39	1,37	1,35	1,34
Permanent fertility	2,76	2,76	2,76	2,76	2,76	2,76	2,76	2,76	2,76	2,76	2,76	2,76	2,76	2,76	2,76	2,76
Instant replacement	2,1	2,09	2,09	2,09	2,09	2,08	2,08	2,08	2,08	2,08	2,08	2,08	2,08	2,07	2,07	2,07
Inertia	2,1	2,1	2,1	2,1	2,1	2,1	2,1	2,1	2,1	2,1	2,1	2,1	2,1	2,1	2,1	2,1
Zero migration	2,62	2,5	2,4	2,31	2,23	2,16	2,1	2,05	2,01	1,97	1,94	1,91	1,89	1,87	1,85	1,84
Constant mortality	2,62	2,5	2,4	2,31	2,23	2,16	2,1	2,05	2,01	1,97	1,94	1,91	1,89	1,87	1,85	1,84
Without changes	2,76	2,76	2,76	2,76	2,76	2,76	2,76	2,76	2,76	2,76	2,76	2,76	2,76	2,76	2,76	2,76

**Table 2:** Births, both sexes.

Option	Region, sub-region, country or area	2020–2025	2025–2030	2030–2035	2035–2040	2040–2045	2045–2050	2050–2055	2055–2060	2060–2065	2065–2070	2070–2075	2075–2080	2080–2085	2085–2090	2090–2095	2095–2100
Evaluations	Kazakhstan	1 253	1 598	1 842	1 631	1 761	1 825	1 959	2 048	1 661	1 240	1 256	1 707	1 913	1 943		
Middle option	Kazakhstan	1 719	1 623	1 685	1 804	1 866	1 837	1 760	1 698	1 678	1 682	1 680	1 651	1 604	1 551	1 514	1 492
High option	Kazakhstan	1 883	1 883	2 036	2 200	2 327	2 381	2 394	2 427	2 501	2 594	2 675	2 728	2 765	2 797	2 848	2 917
Low option	Kazakhstan	1 555	1 363	1 333	1 409	1 420	1 338	1 209	1 092	1 019	976	935	879	810	738	680	636
Permanent fertility	Kazakhstan	1 813	1 793	1 942	2 163	2 338	2 423	2 462	2 534	2 674	2 854	3 028	3 169	3 290	3 422	3 586	3 779
Instant replacement	Kazakhstan	1 374	1 358	1 468	1 623	1 684	1 633	1 550	1 501	1 519	1 570	1 602	1 595	1 566	1 543	1 542	1 558
Inertia	Kazakhstan	1 377	1 362	1 475	1 631	1 693	1 643	1 560	1 511	1 530	1 583	1 615	1 608	1 579	1 556	1 556	1 572
Zero migration	Kazakhstan	1 719	1 623	1 685	1 804	1 866	1 837	1 760	1 698	1 678	1 682	1 680	1 651	1 604	1 551	1 514	1 492
Constant mortality	Kazakhstan	1 719	1 622	1 683	1 801	1 862	1 831	1 751	1 686	1 664	1 665	1 659	1 628	1 578	1 522	1 482	1 457
Without changes	Kazakhstan	1 812	1 793	1 940	2 160	2 332	2 414	2 450	2 517	2 651	2 825	2 991	3 124	3 236	3 358	3 511	3 691

### 4.1 Population size

In the demographic scenario, it is necessary to take into account that the mortality observed in 2020–2021 due to COVID-19 was quite high. The mortality rate observed during this period was different for different age categories, a higher mortality rate was observed in older age groups. The birth rate data for Kazakhstan is decreasing until 2030, then there is a slight increase in the birth rate until 2080, then the birth rate changes the trend to a slight decrease until 2100. Nevertheless, stable population growth is expected in the Republic of Kazakhstan, by 2030 the population is projected at 21,023,114 people, by 2050 the population will reach 25,497,599 people, by 2100 the population is expected to grow to 34,052,098 people. In general, the population of the Republic of Kazakhstan will increase by 6.4 million people by 2050. By 2100, the population is expected to increase by 14.95 million people.

### 4.2 The population structure

By 2030, the male population in Kazakhstan will reach 10,225,311 people, while women - 10,902,910 people. Thus, the ratio of the number of men per 100 women will be 93.8. By 2050, the male population in Kazakhstan will reach 12,546,132 people, while women are expected to be 13,063,845 people. Thus, the ratio of the number of men per 100 women reaches 96. By 2100, the male population in Kazakhstan will reach 16,985,181 people, while 17,108,149 people are expected to be women. Thus, the ratio of the number of men per 100 women will increase to 99.3. According to the obtained data, a stable increase in the dynamics of the ratio of the number of men per 100 women is expected.

The population aged 0 to 14 years will reach 5.87 million people by 2030, and the population aged 15 to 59 years will reach 12.29 million people, while the population over the age of 60 is expected to reach 2.97 million people. The population aged 0 to 14 years will reach 6.55 million people by 2050, while 14.68 million people aged 15 to 59 years, while the population aged 60 + years is expected to reach 4.37 million people. Thus, within twenty years (2030–2050), an increase in the youth population (from 0 to 14 years) is expected by 680,000 people. At the same time, the number of adults aged 15 to 59 will increase by 2.4 million people. The number of elderly population (60+) will increase by 1.4 million people.

### 4.3 Demographic dynamics and elements

It is expected that on January 1, 2030, the fertility rate (the number of births per woman) will be 2.84, and the number of newborns will reach 386,000 people. It is expected that by 2050 the birth rate will be 2.42, the number of newborns - 450,000 people. By 2100, the birth rate will be 1.91, the number of newborns - 401,000 people. Data on the fertility rate show that Kazakhstan is in the second phase, corresponding to a gradual decrease in the fertility level to a value below 2.0 (Table 1).

The mortality rate by 2030 will be 195,000 people, out of 91,000 men and 84,000 women, the average life expectancy at birth is 72.9 years, while the average life expectancy for men will be 68.8 years; for women - 76.7 years. The mortality rate by 2050 will be 255,000 people, out of 114,000 men and 111,000 women, the average life expectancy at birth is 75.4 years, while the average life expectancy for men will be 71.6 years; for women - 79.2 years. The mortality rate by 2100 will be 318,000 people, out of 163,000 men and 156,000 women, the average life expectancy at birth is 82.6 years, while the average life expectancy for men will be 80.2 years; for women - 85.1 years.

The infant mortality rate by 2030 will be 6.4 per 1000 live births. By 2050, the value of this coefficient will decrease to 4.1, followed by a decrease to 2.0 by 2100.

Thus, a stable increase in average life expectancy is expected for the population as a whole, including for men and women. At the same time, the average life expectancy for men will increase by 2.8 years for the period from 2030 to 2050; while, for the period from 2050 to 2100, this indicator will increase by 8.6 years.

The average life expectancy for women will increase by 2.5 years over the period from 2030 to 2050; while, over the period from 2050 to 2100, the average life expectancy for women will increase by 5.9 years. Population growth is expected mainly due to the birth rate. Natural population growth in 2022–2044 (the number of births - the number of deaths) will grow from 205,000 to 237,000 people. Starting from 2045, the dynamics of natural population growth becomes negative and reaches the value of 82.5 thousand people by 2100 (Table 2).

**Fig. 2:**
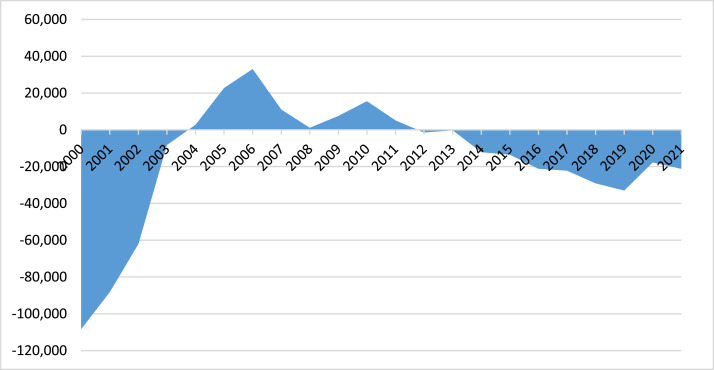
Balance of migration of the population of Kazakhstan.

*Migration.* There are several major migration waves in the history of independent Kazakhstan (Fig. 2). Thus, the 1990s were characterized by mass immigration, only in 1991 the negative balance of migration of the republic amounted to 59,140 people. During this period, the negative balance of migration exceeded the natural population growth by more than 2 times, which decreased in the 1990s by almost 2 times, due to a sharp decrease in the number of births and an increase in the number of deaths compared to the previous decade [5].

After the increase in immigration noted in the 2000s, Kazakhstan has seen a repeated increase in the rate of net emigration since 2012. The number of immigrants has decreased by an average of 12.2% annually since 2005, while the number of emigrants has grown by 9.6% since 2015. If 30 thousand citizens left the country in 2015, then by the end of 2019 the indicator increased by 30% and amounted to 45,2 thousand people, which was the largest number in the last 10 years. In general, over the past ten years, more than 366 thousand people have irretrievably left the country. The pandemic has slowed down the pace of migration somewhat, but in 2021, 3256 people left the country, among them 22,325 people of working age.

### 4.4 The long-term population forecast

**Fig. 3:**
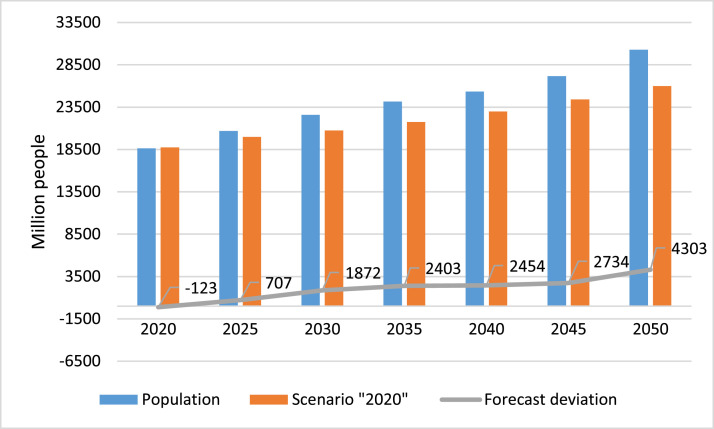
Long-term population forecast, scenario “2020” and deviation to “2020”.

The long-term population forecast is presented in Fig. 3. The population of Kazakhstan in 2020 is 18,628 thousand people. In 2030, the population according to the forecast will be 22,599 thousand people, in 2050 it will be 30,292 thousand people. According to the forecast, over 30 years the population may increase by 7.6 million people or by 34%. In the “2020” scenario, the population in 2025 will be 19,982 thousand people, in 2040 it will increase by 15% and amount to 22,980 thousand people. In 2050, according to the “2020” scenario, the population will be 25,984. To 2020, the “2020” scenario shows an increase in the population by 39.4% by 2050. It turns out that in 30 years the population can increase by 7.3 million people.

The population forecast deviation from the “2020” scenario in different years shows a different trend. In 2030, the deviation is 1.8 million people, in 2035, the deviation in the forecast is 2.4 million people. In 2050, the deviation is 4.3 million. Therefore, in the forecast, the deviation has to be and affects the forecast period in direct proportion. Namely, the longer the forecast period is, the deviation occurs more.

Table 3 shows the population forecast by age group until 2050. The population size decreases as the age increases as the age decreases. Namely, according to official statistics, in 2020, children 0–4 years old makeup 1,014,153 people, children 5–9 years old make up 957,417 people, children over 10 years old to 14 years old make up 793,518 people, then up to 39 years old the population is more than the population from 44 years old. The older the age of the population, the smaller their number by age group. Based on 2020, a forecast was made until 2050. According to the forecast, all age groups have an increasing trend. Thus, the number of children from 0 to 19 years is 8,039,203 people. And the number of the group from 80 to 100 years will be 2.3 million people.

**Table 3:** Population forecast by age groups of the population of Kazakhstan until 2050, people.

Age	2020	2025	2030	2035	2040	2045	2050
0–4	1,014,153	2,090,947	2,070,290	2,056,442	2,111,544	2,349,709	2,919,359
05–09	957,417	961,164	1,981,695	1,962,117	1,948,993	2,001,216	2,226,937
10–14	793,518	793,576	796,681	1,642,571	1,626,344	1,615,466	1,658,752
15–19	604,435	606,218	606,262	608,635	1,254,863	1,242,466	1,234,155
20–24	591,521	593,978	595,730	595,774	598,105	1,233,154	1,220,971
25–29	745,710	756,671	759,814	762,055	762,111	765,093	1,577,444
30–34	787,467	803,484	815,294	818,680	821,095	821,155	824,369
35–39	658,488	657,402	670,774	680,633	683,460	685,476	685,527
40–44	571,200	580,479	579,522	591,309	600,000	602,493	604,270
45–49	522,236	530,967	539,592	538,702	549,659	557,738	560,055
50–54	466,797	475,150	483,094	490,942	490,132	500,101	507,452
55–59	461,380	473,496	481,970	490,027	497,987	497,166	507,279
60–64	344,396	364,611	374,186	380,882	387,250	393,541	392,892
65–69	231,939	251,304	266,055	273,042	277,928	282,575	287,165
70–74	130,760	141,997	153,853	162,883	167,161	170,152	172,997
75–79	71,036	79,680	86,527	93,752	99,255	101,861	103,684
80–84	58,015	64,241	72,058	78,250	84,783	89,760	92,117
85–89	15,924	17,360	19,223	21,562	23,415	25,370	26,859
90–94	5346	5465	5958	6597	7400	8036	8707
95–99	1698	1196	1223	1333	1476	1656	1798
100+	698	473	333	340	371	411	461

**Fig. 4:**
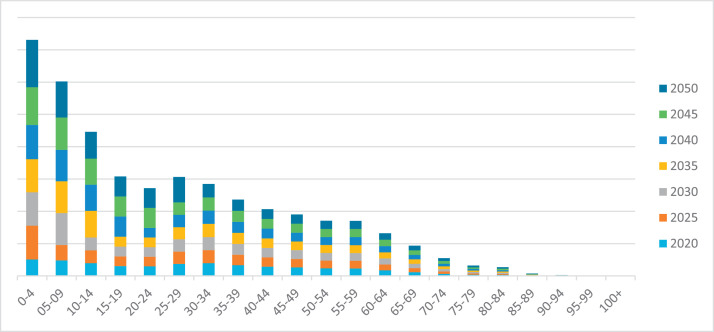
Population forecast by age groups in Kazakhstan until 2050.

Fig. 4 shows the growth trend in increasing age groups. For example, the number of children from 0 to 4 to 2050 will increase 3 times, and the number of the group from 70 to 74 years will increase by only 32%. The number of young people (15–29 years old) entering the labor market will be more than 4 million.

Thus, this observation can be explained by several reasons:

- the birth rate of the population is increasing every year;
- the mortality rate of the population increases every year;
- the reproductive capacity of the population decreases at a more mature age, etc.

### 4.5 Forecast based on alternative methods

According to the results of the calculations presented in Tables 4 and 5, the alternative forecasting methods used gave different forecast values of the population by 2050. The forecast based on three methods – the method of average growth coefficients, exponential curve, natural and mechanical movement of the population – showed that the population of Kazakhstan will exceed 25 million people.

**Table 4:** Forecasts of population on the methods of prospects and movements.

Year	Forecasting methods				
	Median	Sub 95%	Top 95%	Perspective	Age shift method
2020	18,776,707	18,776,707	18,776,707	18,631,779	18,631,779
2025	19,787,745	19,592,923	19,992,639	19,981,164	20,688,846
2030	20,639,019	20,203,047	21,101,999	21,395,654	22,598,690
2035	21,483,455	20,749,858	22,311,954	22,851,782	24,150,340
2040	22,370,403	21,263,294	23,658,304	24,349,548	25,434,411
2045	23,242,950	21,721,057	25,085,662	25,888,953	27,161,479
2050	24,024,036	22,044,842	26,530,786	27,469,995	30,287,421
2055	24,681,909	22,187,089	27,945,128		
2060	25,243,435	22,186,056	29,352,979		
2065	25,751,107	22,105,199	30,912,456		
2070	26,223,002	21,987,400	32,601,034		
2075	26,656,354	21,788,522	34,339,559		
2080	27,041,822	21,556,338	36,202,912		
2085	27,376,705	21,229,101	38,199,934		
2090	27,649,907	20,862,931	40,217,374		
2095	27,839,349	20,432,742	42,361,479		
2100	27,917,815	19,822,646	44,408,214

**Table 5:** Forecasting using the average growth coefficient, exponential curve formulas, natural and mechanical movements, second and third degree polynomials.

Year	Forecasting methods				
	Average growth rate	Exponential curve	Natural and mechanical movement of the population	Polynomial of the 2nd degree	Polynomial of the 3rd degree
2020	18,631,779	18,631,779	18,631,779	18,631,779	18,631,779
2025	19,582,187	19,587,051	19,650,140	24,366,872	23,294,862
2030	20,581,075	20,591,300	20,724,162	27,312,022	25,145,461
2035	21,630,917	21,647,039	21,856,887	30,827,572	26,950,009
2040	22,734,311	22,756,906	23,051,524	34,913,522	28,582,701
2045	23,893,990	23,923,678	24,311,456	39,569,872	29,917,732
2050	25,112,823	25,150,271	25,640,252	44,796,622	30,829,297

Close forecast values are obtained based on the methods of movement (Table 4) and a polynomial of the third degree (Table 5). According to them, the population will reach 30 million people.

The intermediate value between the two above-mentioned variants of forecasts was given by the perspective method (Table 4). According to it, the population will reach 27 million people by 2050.

The highest predicted values were shown by the method based on a polynomial of the second degree (Table 5). Here the predicted values of the population amounted to more than 40 million people or a doubling of the population. However, such a forecast is unlikely.

Based on the results of comparing the forecast values obtained using the method of movement and extrapolation, it can be concluded that both methods showed fairly average accuracy. The method of forecasting shifts is simple and undemanding to the amount of data, while it is very sensitive to a sharp change in the predicted indicators. The extrapolation method is demanding for the uniformity and comparability of the predicted data.

Demographic forecasting is a fundamental element for developing effective country development strategies. Understanding future population changes allows proactive measures to be taken to ensure sustainable economic, social, and environmental development.

- Economic development, in particular:

Labor Market: Labor force forecasting helps governments, companies, and educational institutions adapt recruitment and training strategies to future needs.

Pension system: Knowledge of demographic trends is necessary for effective pension systems management, taking into account the change in the ratio of the working-age and retired population.

- Social sphere, which includes:

Healthcare: Forecasting population structure and morbidity influences the planning of medical infrastructure and resources.

Education: Determination of future educational needs based on the number of schoolchildren and students.

- Demographic forecasting is used for political management on the following issues:

Migration: Forecasting demographic changes helps shape migration policies, including labor migration and refugee management.

Social stability: Knowledge of population dynamics helps prevent social tensions associated with demographic changes.

- In addition, population projections are important for estimating resource consumption and developing sustainable natural resource use strategies. Environmental planning requires adapting urban planning and development strategies to meet expected population growth.

## Limitations

None.

## Ethics Statement

We did not conduct human or animal studies in this research.

The statistical data used are in the public domain.

## CRediT authorship contribution statement

**Lazat S. Spankulova:** Conceptualization, Data curation, Formal analysis, Funding acquisition, Investigation, Methodology, Project administration, Resources, Software. **Zaure K. Chulanova:** Conceptualization, Formal analysis, Investigation, Methodology, Supervision, Validation, Visualization, Writing – original draft, Writing – review & editing.

## Data Availability

Demograph Forecast (Original data) (Mendeley Data).
